# Morroniside ameliorates glucocorticoid-induced osteoporosis and promotes osteoblastogenesis by interacting with sodium-glucose cotransporter 2

**DOI:** 10.1080/13880209.2023.2173787

**Published:** 2023-02-14

**Authors:** Hou-Zhi Yang, Runbei Dong, Yutao Jia, Yuqiao Li, Gan Luo, Tianhao Li, Yao Long, Shuang Liang, Shanshan Li, Xin Jin, Tianwei Sun

**Affiliations:** a Tianjin Medical University, Tianjin, China; b School of Medicine, Nankai University, Tianjin, China; c Department of Spinal Surgery, Tianjin Union Medical Center, Tianjin, China

**Keywords:** MC3T3-E1 cells, zebrafish, molecular docking, glucose pockets, SGLT2

## Abstract

**Context:**

Morroniside (MOR) possesses antiosteoporosis (OP) effects, but its molecular target and relevant mechanisms remain unknown.

**Objective:**

We investigated the effects of MOR on glucocorticoid-induced OP and osteoblastogenesis and its underlying mechanisms.

**Materials and methods:**

The effects of MOR (10–100 μM) on the proliferation and differentiation of MC3T3-E1 cells were studied *in vitro*. The glucocorticoid-induced zebrafish OP model was treated with 10, 20 and 40 μM MOR for five days to evaluate its effects on vertebral bone density and related osteogenic markers. In addition, molecular targets prediction and molecular docking analysis were carried out to explore the binding interactions of MOR with the target proteins.

**Results:**

In cultured MC3T3*-*E1 cells, 20 μM MOR significantly increased cell viability (1.64 ± 0.12 vs. 0.95 ± 0.16; *p* < 0.01) and cell differentiation (1.57 ± 0.01 vs. 1.00 ± 0.04; *p* < 0.01) compared to the control group. MOR treatment significantly ameliorated vertebral bone loss in the glucocorticoid-induced OP zebrafish model (0.86 ± 0.02 vs. 0.40 ± 0.03; *p* < 0.01) and restored the expression of osteoblast-specific markers, including ALP, Runx2 and Col-І. Ligand-based target prediction and molecular docking revealed the binding interaction between MOR and the glucose pockets in sodium-glucose cotransporter 2 (SGLT2).

**Discussion and conclusions:**

These findings demonstrated that MOR treatment promoted osteoblastogenesis and ameliorated glucocorticoid-induced OP by targeting SGLT2, which may provide therapeutic potential in managing glucocorticoid-induced OP.

## Introduction

Osteoporosis (OP) is a metabolic disease leading to decreased bone strength and susceptibility to fragility fractures. OP is generally classified into two broad categories: primary OP and secondary OP. Glucocorticoid-induced OP is the most common cause of secondary and iatrogenic OP in individuals under 50 years old (Compston et al. 2019). Its incidence rate has increased progressively due to the rising use of glucocorticoid*-*containing medications (Compston 2018). Pathological fractures and the associated medical expenses have imposed a substantial economic burden on society and individuals (Shoback et al. 2020). Furthermore, OP patients suffer from long treatment cycles, severe side effects and recurrence after discontinuation. Therefore, it is urgent to develop new interventions for this intractable disease (Gupta and Aronow 2007).

*Corni Fructus* (CF) is a traditional Chinese medicine derived from the dry ripe sarcocarp of *Cornus officinalis* Sieb. et Zucc. (Cornaceae) which has been widely used clinically to treat diseases such as diabetic nephropathy, tuberculosis and OP (Yamabe et al. 2007; Lee et al. 2012). The iridoid monoterpenoid glycoside constituents from CF have shown numerous pharmacological activities, including antioxidant activity (Yokozawa et al. 2010), hypoglycemic activity (Hsu et al. 2006; He 2011), nephroprotective activity (Xu and Hao 2004), myocardial protection activity (Pi et al. 2017) and anti-osteoporotic effects (Sun et al. 2013; Bellavia et al. 2021). Furthermore, studies have demonstrated that CF-comprised traditional Chinese formulas could effectively promote osteogenic differentiation and attenuate OP progression (Zhang et al. 2019; Xia et al. 2020). Morroniside (MOR) is a major bioactive iridoid glycoside in CF (Ye et al. 2009). It has various pharmacological benefits, such as promoting vascular regeneration (Wang et al. 2019), reducing infarct volume in the brain (Liu et al. 2016), inhibiting platelet aggregation (Liu et al. 2020), promoting neuronal cell proliferation and differentiation (Zeng et al. 2018), protecting cardiomyocytes (Lee et al. 2017), protecting kidney cells (Pi et al. 2017) and improving glucose homeostasis (Xu et al. 2004). In addition, recent studies have demonstrated that MOR has promising anti-OP bioactivities such as enhancing osteoblast formation (Lee et al. 2021) and decreasing osteoclast differentiation (Liu et al. 2021), but the mechanism behind its action remains unclear.

Osteoblasts are critical cells in bone formation to promote bone synthesis and mineralization, playing a significant role in bone formation (Jilka et al. 2014). In osteoporotic patients, decreased proliferation and differentiation of osteoblasts are the most common pathological processes (Lee et al. 2017). Therefore, promoting the proliferation and differentiation of osteoblasts is critical in preventing and treating OP. Although significant progress in exploring the anti-OP effects of MOR has been made, the molecular targets and the mechanisms underlying MOR functions remain largely unknown. Here, we established a glucocorticoid-induced OP zebrafish model to evaluate the anti-OP function of MOR. We demonstrated that MOR could improve glucocorticoid-induced OP and promote osteoblast proliferation and differentiation. Mechanistically, its action may be associated with SGLT2 interaction. This study provides new insight into the pharmacological mechanism of MOR and reveals a potential therapeutic target for glucocorticoid-induced OP.

## Materials and methods

### Preosteoblastic cell line

MC3T3-E1 cells (Procell Life Science & Technology Co., Ltd., Wuhan, China) were cultured in a complete medium, including high glucose 90% aMEM (Procell Life Science & Technology Co., Ltd., Wuhan, China), 10% foetal bovine serum (Gibco, Carlsbad, CA) and 1% triple antibiotic (penicillin, streptomycin and amphotericin) (Solarbio, Beijing, China) at 37 °C in a humid environment with 5% CO_2_, and the medium was replaced every two days. When cells were 80–90% confluent, the cell medium was replaced with an induction medium, which was complete media including 50 mg/mL l-ascorbic acid 2-phosphate and 5 mM β-glycerophosphate.

### Cell proliferation assay

MC3T3-E1 cells were seeded in 96-well plates at 5000 cells/well. After 24 h of incubation, cells were exposed for 24 h to various concentrations of MOR (0, 10, 20, 40, 80 and 100 µM). Subsequently, 10 µL of CCK-8 (5.0 mg/mL) was added to each well and incubated at 37 °C for another 2 h. The absorbance value (OD) was measured at 450 nm on a microplate spectrophotometer (Bio-Rad Model 680, Hercules, CA).

### Alizarin red staining

After 14 days of induction, the MC3T3-E1 cells were fixed in 4% PFA and rinsed twice with ddH_2_O, followed by 1 mL of 95% ethanol dripping into each well. The cells were air-dried, then covered with alizarin red staining (ARS) solution (1%, pH 4.2) for 30 min. The dye was then removed and rinsed with ddH_2_O.

### Alkaline phosphatase staining

After 14 days of induction, MC3T3-E1 cells were washed with phosphate-buffered saline (PBS) and fixed with 4% PFA for 30 min at room temperature. After fixation, cells were washed with PBS three times. ALP staining solution (Beyotime Biotechnology, Shanghai, China, C3206) was then added and incubated at room temperature under dark conditions for 30 min. The activity of the MOR groups was normalized according to the control group.

### Quantitative real-time PCR

Total RNA was extracted using TRIzol (Invitrogen, Carlsbad, CA) and reverse transcribed using the PrimeScript RT Reagent Kit (TaKaRa Bio, Kusatsu, Japan). Each qRT-PCR included 2 µg of RNA, 1 µL of oligo (dt)/RT-primer, and nuclease-free water up to 20 µL. The qRT-PCR cycles were 42 °C for 60 min and 70 °C for 5 min. Then, cDNA samples were used as a template for real-time qPCR. The real-time qPCR system included 1 L of cDNA, 0.2 µL of forward and reverse primers, 5 µL of SYBR Green I Master (ROX), and nuclease-free water up to 10 µL. Real-time qPCR cycles were set to 95 °C for 30 s, 95 °C for 5 s, 55 °C for 10 s and 72 °C for 10 s, and those conditions were repeated for 40 cycles. The 2^–ΔΔCT^ method was utilized to analyse the data. The primer sequences are listed in Table 1.

**Table 1.** The primer sequences of genes for PCR analysis.

Gene	Forward primer (5′–3′)	Reverse primer (5′–3′)
P21 (*Mus musculus*)	ATGTCCAATCCTGGTGATGTC	GAAGTCAAAGTTCCACCGTTC
Ccnd1 (*Mus musculus*)	CGTATCTTACTTCAAGTGCGTG	ATGGTCTCCTTCATCTTAGAGG
PCNA (*Mus musculus*)	GAAGTTTTCTGCAAGTGGAGAG	CAGGCTCATTCATCTCTATGGT
ALP (*Mus musculus*)	TCATTCCCACGTTTTCACATTC	GTTGTTGTGAGCGTAATCTACC
Col-1 (*Mus musculus*)	TGAACGTGGTGTACAAGGTC	CCATCTTTACCAGGAGAACCAT
BMP-2 (*Mus musculus*)	AGTAGTTTCCAGCACCGAATTA	CACTAACCTGGTGTCCAATAGT
Runx2 (*Mus musculus*)	CCTTCAAGGTTGTAGCCCTC	GGAGTAGTTCTCATCATTCCCG
OSX (*Mus musculus*)	GACTACCCACCCTTCCCTCACTC	TAGACACTAGGCAGGCAGTCAGAC
ACTB (*Danio rerio*)	GTGATGGACTCTGGTGATGGTGTG	CACGCTCGGTCAGGATCTTCATC
Col-1 (*Danio rerio*)	CCAACGGCTTCCAGTTCCAGTATG	TCCATGTAGGCGATGCTGTTCTTG
Runx2 (*Danio rerio*)	AGACTCCGACCTCACGACAACC	GGCAGCACCGAGCACAGAAAG
OPN (*Danio rerio*)	TGGGCGGCTTGACATTTGTGAG	TCTATCTCTGAGGTGCTGGTGTTCC
OSX (*Danio rerio*)	AGGCTTGCTAACACCAACTGGAAG	GGGAAACACTGGAGGTCTGGAAAG

### Western blotting

The RIPA method was utilized to isolate total protein. Lysates were centrifuged at 13,000 rpm for 15 min at 4 °C. The supernatant was obtained. After adding 4 × SDS-PAGE loading buffer to the supernatant (supernatant: loading buffer = 3:1), the mixture was mixed and heated at 100 °C for 10 min. The optimum concentration of SDS-PAGE was used to separate the total protein sample. After electrophoresis, the protein was transferred to a PVDF membrane (Merck Millipore, Burlington, MA) in an ice bath with 360 mA current for one hour and 45 min. The membrane was soaked in 5% skim milk for two hours and then incubated in primary antibody (TBST:antibody = 800:1) at 4 °C overnight and IgG-HRP (TBST:IgGHRP = 8000:1) at room temperature for 2 h. After washing with TBST, the membrane was visualized by enhanced chemiluminescence substrate (Cell Signaling Technology, Boston, MA) and a chemiluminescence detection system (Promega, Madison, WI). GAPDH served as an internal reference.

### Glucocorticoid-induced zebrafish OP model and MOR treatments

Wild-type zebrafish (AB strain) farming was conducted according to the standard procedure. Adult zebrafish mate and spawn naturally under the condition of a 10/14 h dark/light cycle. The zebrafish embryos and larvae were cultured in E3 medium solution (5.0 mM NaCl, 0.17 mM KCl, 0.33 mM CaCl_2_ and 0.33 mM MgSO_4_) under isothermal conditions at 28 °C. The use of zebrafish in this study met the requirements of the institutional Ethical Guidelines for Animal Experiments (ethics approval number: 2022-SYDWLL-000052). Synchronized embryos were placed into a six-well plate at three days postfertilization (dpf). Zebrafish larvae were then exposed to 75 µg/mL prednisone and different concentrations of MOR (0, 10, 20 and 40 µM). Next, zebrafish larvae were exposed to 25 µg/mL of prednisone (Wang et al. 2018), 20 µM of MOR, and SGLT2 inhibitor (1 µM). At the same time, zebrafish only treated with the medium solution served as a normal control group. All these groups were incubated at 28 °C until 8 dpf. The medium solution was changed every other day.

### Bone matrix alizarin red S labelling

Alizarin red S (AR-S), a fluorescent dye, could bind with a mineralized nodule in the bone matrix. AR-S labelling could reflect the degree of bone mineralization. Zebrafish larvae aged 8 dpf were dyed with 0.2% AR-S solution for 2 h. Then, they were washed with E3 water for 15 min on a shaking table at 50 rpm (repeated three times). Fluorescence images of the AR-S-stained vertebrate column were captured under an AZ100 fluorescence microscope (Nikon, Tokyo, Japan). The integral optical density of vertebrae was measured by ImageJ analysis software (Bethesda, MD).

### Behavioural trajectory analysis of zebrafish larvae

Zebrafish larvae from different treatment groups were placed in a 24-well plate. First, the filling/draining switch was turned on and filled with pure water. The filling was stopped when there were no air bubbles in the tube and water flowed out of the overflow tube. Next, the heating/cooling switch was turned on, the water temperature was increased to 28 °C, and then the test was started for 20 min. The 24-well plate was tilted into the machine to avoid air bubbles and let the fish adapt for 15 min before starting the test. After 10 min of testing, the total moving distance, the time required to move to the centre point, and the trajectory and thermal graphs were recorded. The behavioural trajectory was measured using EthoVision XT (version 10; Noldus, Wageningen, The Netherlands) motion tracking software.

### Target prediction and molecular docking

In order to predict the targets of MOR, the web-based target prediction tools TargetNet (Yao et al. 2016), Swiss*TargetPrediction* (Daina et al. 2019) and Similarity Ensemble Approach (*SEA*) (Keiser et al. 2007) were used to identify molecular targets.

The structure SDF file of compound MOR was downloaded from the PubChem database (https://pubchem.ncbi.nlm.nih.gov/) and converted into a PDB file using Open Babel 2.3.2 software. The three-dimensional structure of SGLT2 was obtained from the AlphaFold Protein Structure database (https://alphafold.ebi.ac.uk/). After removing all water molecules and ligands in SGLT2 using PyMOL software, the SGLT2 structure was further modified by hydrogenation and charge balance using AutoDockTools software. Finally, the molecular docking of SGLT2 and MOR was performed using AutoDock Vina 1.1.2. The genetic algorithm was employed for the docking process, and the lowest score was visualized using PyMOL.

### Statistical analysis

Data from each group are shown as the mean ± SD. All data are expressed as the mean ± SEM (standard error). Mean values, standard deviations and significant differences were examined with GraphPad Prism8 (La Jolla, CA). Comparisons of data were acquired by one-way ANOVA followed by Bonferroni’s post hoc test. *p* < 0.05 was considered statistically significant.

## Results

### Morroniside promoted the proliferation and differentiation of MC3T3-E1 cells

**Figure 1.**
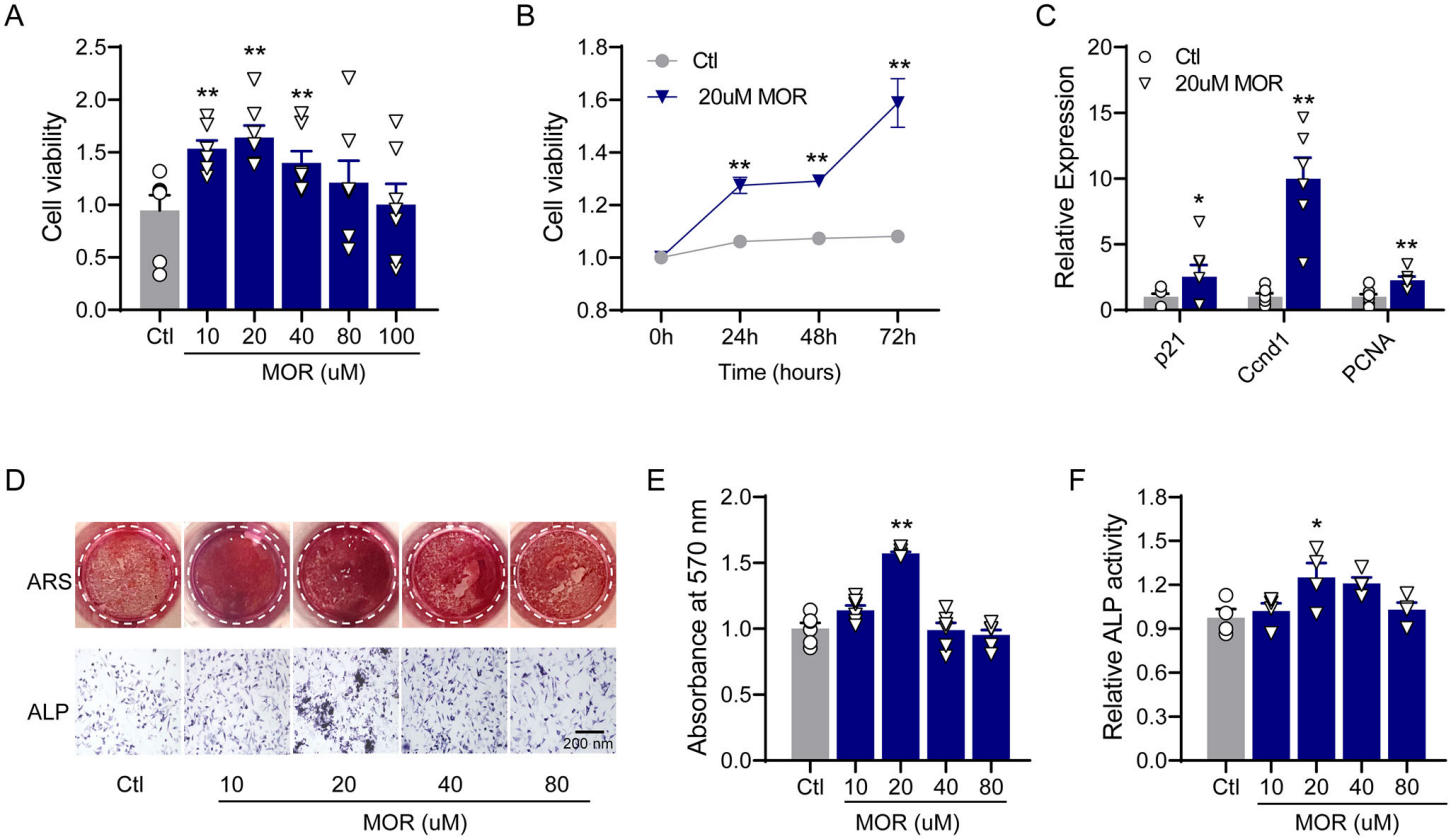
Morroniside promotes the proliferation and differentiation of MC3T3-E1 cells. (A) Cell viability was measured after treatment with different concentrations of morroniside (10, 20, 40 and 80 μM). (B) After 20 μM of MOR treatment, cell viability was monitored over 72 h, and a time*-*dependent increase in cell viability was observed. (C) The expression of p21, Ccnd1 and PCNA was determined by RT-PCR. (D) The effects of MOR on MC3T3-E1 cell differentiation and alkaline phosphatase (ALP) activity as shown by alizarin red staining (ARS) and ALP staining. Quantitative analysis of ARS (E) and ALP stating (F). *p< 0.05, **p< 0.01 vs. Ctl. *n* = 4–6.

To investigate the effects of MOR on the proliferation of osteoblast cells, we treated MC3T3-E1 cells with MOR at concentrations of 10–100 µM. As shown in Figure 1(A), after 24 h of intervention, cell viability was significantly higher in the MOR treatment groups than in the control group. Furthermore, we found that 20 µM of MOR was the most effective concentration for increasing the viability of MC3T3-E1 cells (Figure 1(A,B)). Dramatically, the effect of 20 µM MOR on the viability of MC3T3-E1 cells was increased after 72 h of exposure (Figure 1(B)). Next, we examined the effects of 20 µM MOR on the expression of cell cycle-related genes. qRT-PCR analysis showed that the expression of p21, CCND1 and PCNA was significantly upregulated in 20 µM MOR-treated MC3T3-E1 cells (Figure 1(C)).

**Figure 2.**
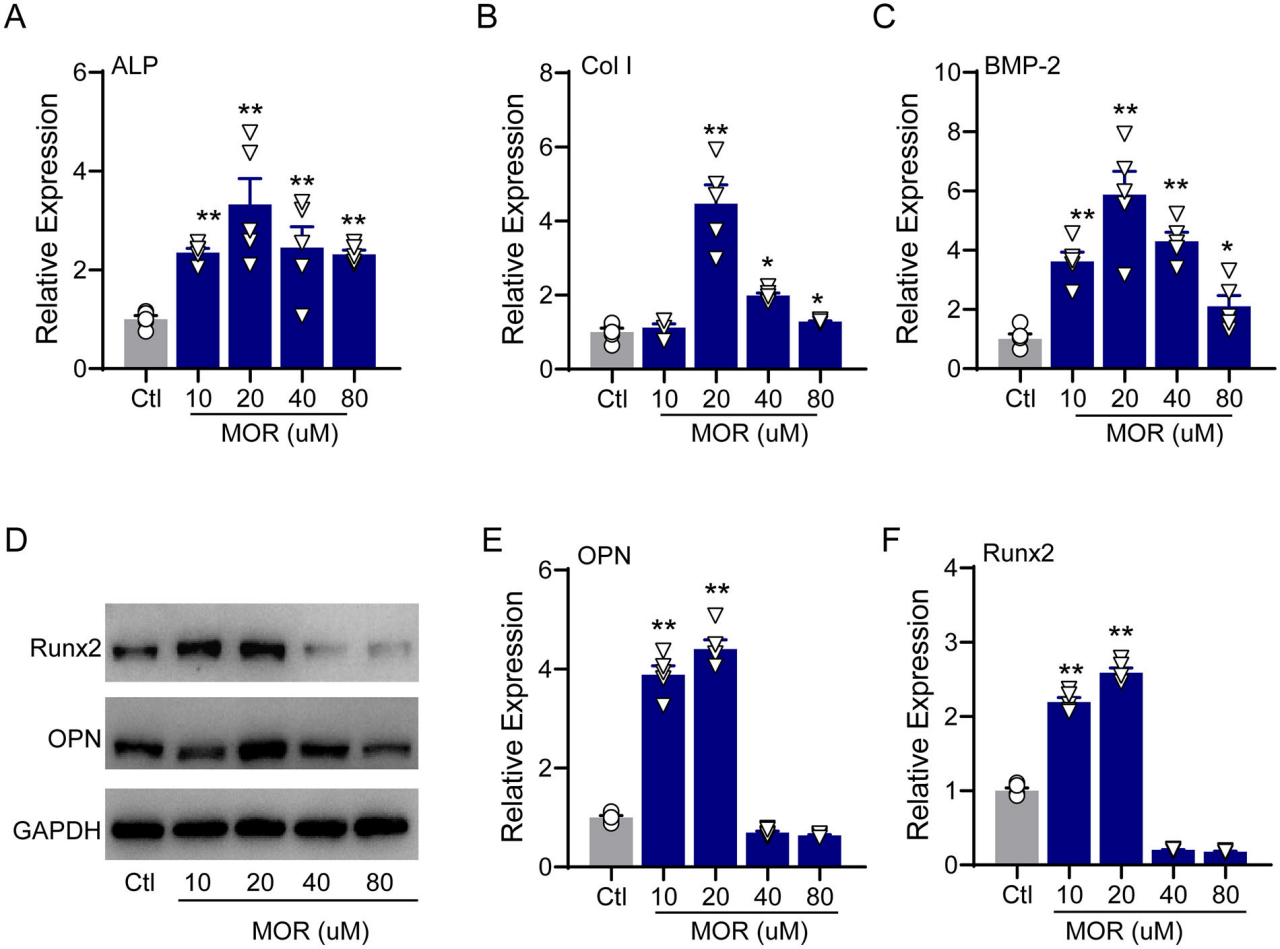
RT-PCR quantification of the osteogenic differentiation marker genes ALP (A), Col I (B) and BMP2 (C) in the control and MOR treatment groups. (D) Runx2, OPN and GAPDH protein levels were detected by Western blotting in MC3T3-E1 cells. NOR (10 and 20 μM) significantly increased OPN (E) and Runx2 (F) protein levels. *p< 0.05, **p< 0.01 vs. Ctl. *n* = 6.

We then evaluated the effects of MOR on osteogenic differentiation and alkaline phosphatase (ALP) activity by using ARS and ALP staining, respectively. After seven days of culture with MOR, ARS and ALP staining demonstrated that 20 µM of MOR significantly increased the differentiation and ALP activity of MC3T3-E1 cells (Figure 1(D,F)). The qRT-PCR results revealed that the mRNA levels of ALP (Figure 2(A)), Col I (Figure 2(B)) and BMP2 (Figure 2(C)) were significantly increased in the MOR treatment groups. Furthermore, compared to the controls, the protein levels of principal osteogenic markers, such as OPN and Runx2, were significantly elevated in the 10 and 20 µM MOR groups (Figure 2(E,F)). These results indicated that 20 µM MOR remarkably promoted the proliferation and differentiation of MC3T3-E1 cells.

### MOR ameliorated glucocorticoid-induced OP and mobility impairment in the zebrafish model

**Figure 3.**
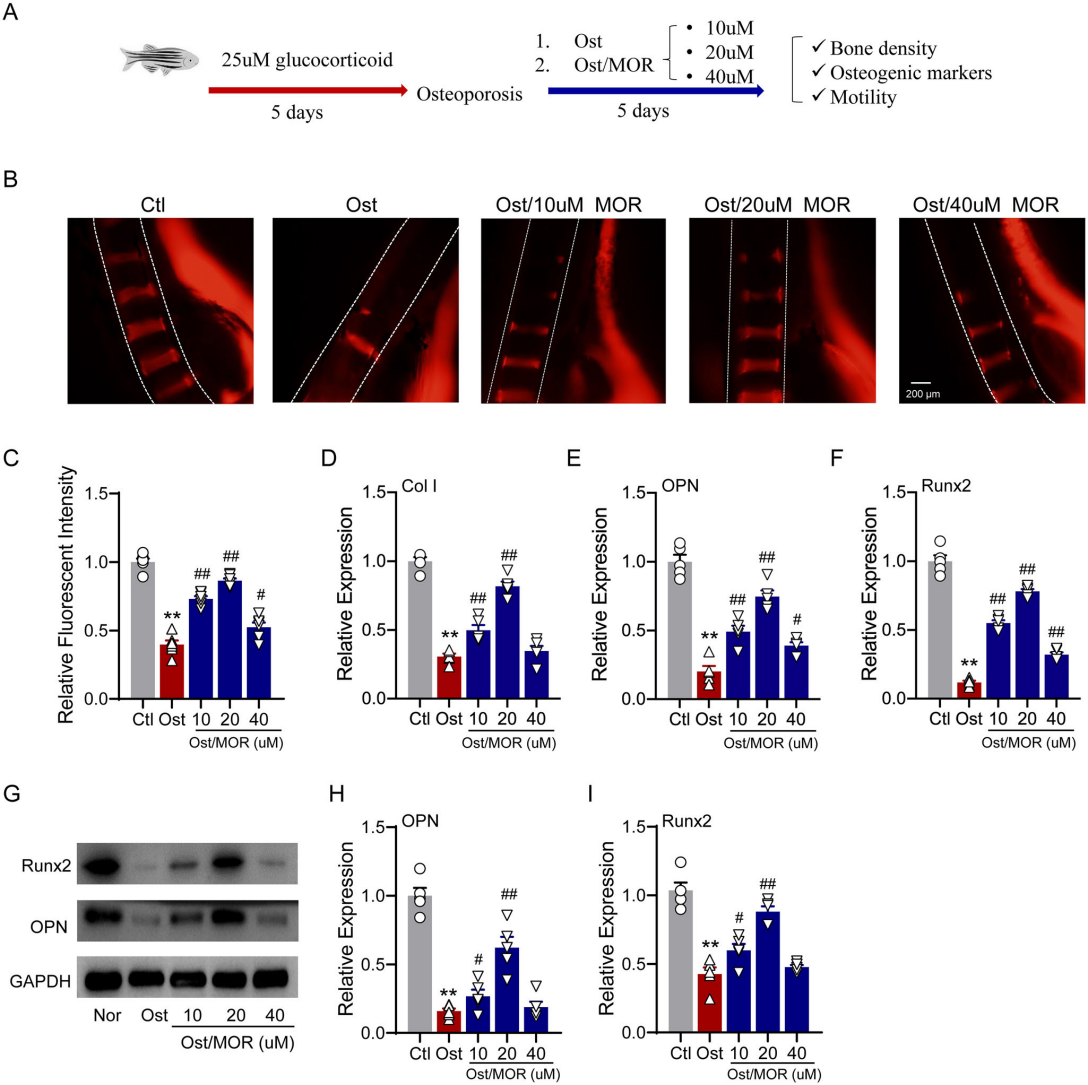
Morroniside ameliorates glucocorticoid-induced OP in a zebrafish model. (A) Schematic illustration of the experimental design for the MOR *in vivo* study in a zebrafish OP model. (B) Representative fluorescence images of normal and MOR-treated zebrafish. (C) Quantification of the fluorescence intensity of the spinal cord column. RT-PCR quantification of osteogenic differentiation marker genes Col I (D), OPN (E) and Runx2 (F) in the normal (Nor), OP (Ost) and MOR treatment groups. (G) Runx2, OPN and GAPDH protein levels were detected by Western blotting in zebrafish. MOR treatments significantly increased the OP-associated decline in OPN (I) and Runx2 (J) protein levels. **p< 0.01 vs. Ctl; ^#^*p*< 0.05, ^##^*p*< 0.01 vs. Ost. *n* = 6.

To assess the anti-OP effects of MOR *in vivo*, we employed a glucocorticoid-induced zebrafish OP model for this study. In the postfertilization stage, the zebrafish larvae were treated with 25 µM of glucocorticoid for five days. After that, we added 10, 20 and 40 µM MOR for five days to observe the changes in vertebral bone density, related osteogenic markers and motility of zebrafish (Figure 3(A)).

As shown in Figure 3(B), glucocorticoids led to a severe decrease in bone density, whereas the ARS showed that vertebral bone mass and vertebral stage number were significantly increased in the 10, 20 and 40 µM MOR groups compared with the OP group (Figure 3(C)). Consistently, 20 µM of MOR treatment showed the most significant effects, as we observed in MC3T3-E1 cells.

In addition, qRT-PCR showed that the expression of osteogenic markers, including OPN, Col I and Runx2, was significantly increased in the MOR groups (Figure 3(D–F)). Furthermore, Western blot analysis showed that OPN and Runx2 expression levels significantly increased with 10 and 20 µM MOR treatment groups (Figure 2(H–J)). We also found that the effect of MOR on the bone mass of zebrafish larvae was concentration-dependent, ranging from 10 to 20 µM.

**Figure 4.**
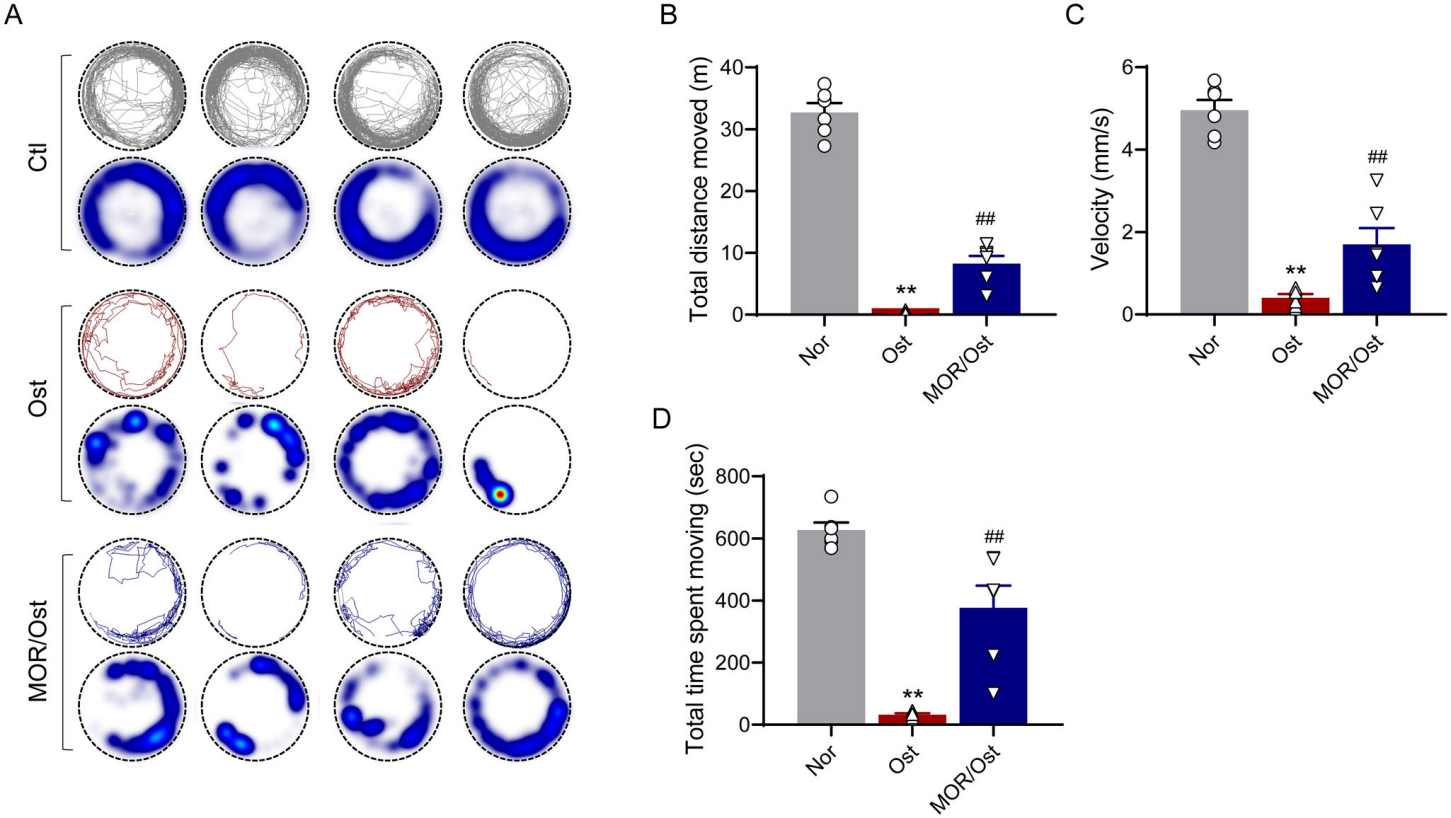
Morroniside ameliorates mobility impairment in OP zebrafish. (A) The locomotive trajectory of normal (Nor), OP (Ost) and MOR-treated zebrafish in 20 min. The lines indicate zebrafish locomotive trajectories. Heatmap colours indicate the duration of time the zebrafish spent in each location over 10 min. MOR treatment significantly increased the total distance moved (B), velocity (C) and total time spent moving (D) in OP zebrafish. **p< 0.01 vs. Ctl; ^##^*p*< 0.01 vs. Ost. *n* = 6.

In elderly patients with OP, the decrease in bone mass and the destruction of bone microstructure can lead to a certain degree of activity limitation in daily life, which can be manifested as reduced or limited motor ability such as difficulty walking or intermittent claudication (Sakai et al. 2020), which can lead to a significant decrease in quality of life in severe cases (Giangregorio et al. 2015). Therefore, based on the above clinical manifestations, we hypothesized that zebrafish larvae might have a similar condition. Next, we used a behavioural tracking system to quantify the mobility of the OP zebrafish model. The behavioural trajectory analysis showed that the total distance travelled, velocity and time spent moving were significantly lower in the osteoporotic group than in the control group (Figure 4(A)). In contrast, 20 µM of MOR treatment markedly improved the locomotor performance in the OP zebrafish model (Figure 4(B–D)).

### Target prediction and molecular docking of morroniside

**Figure 5.**
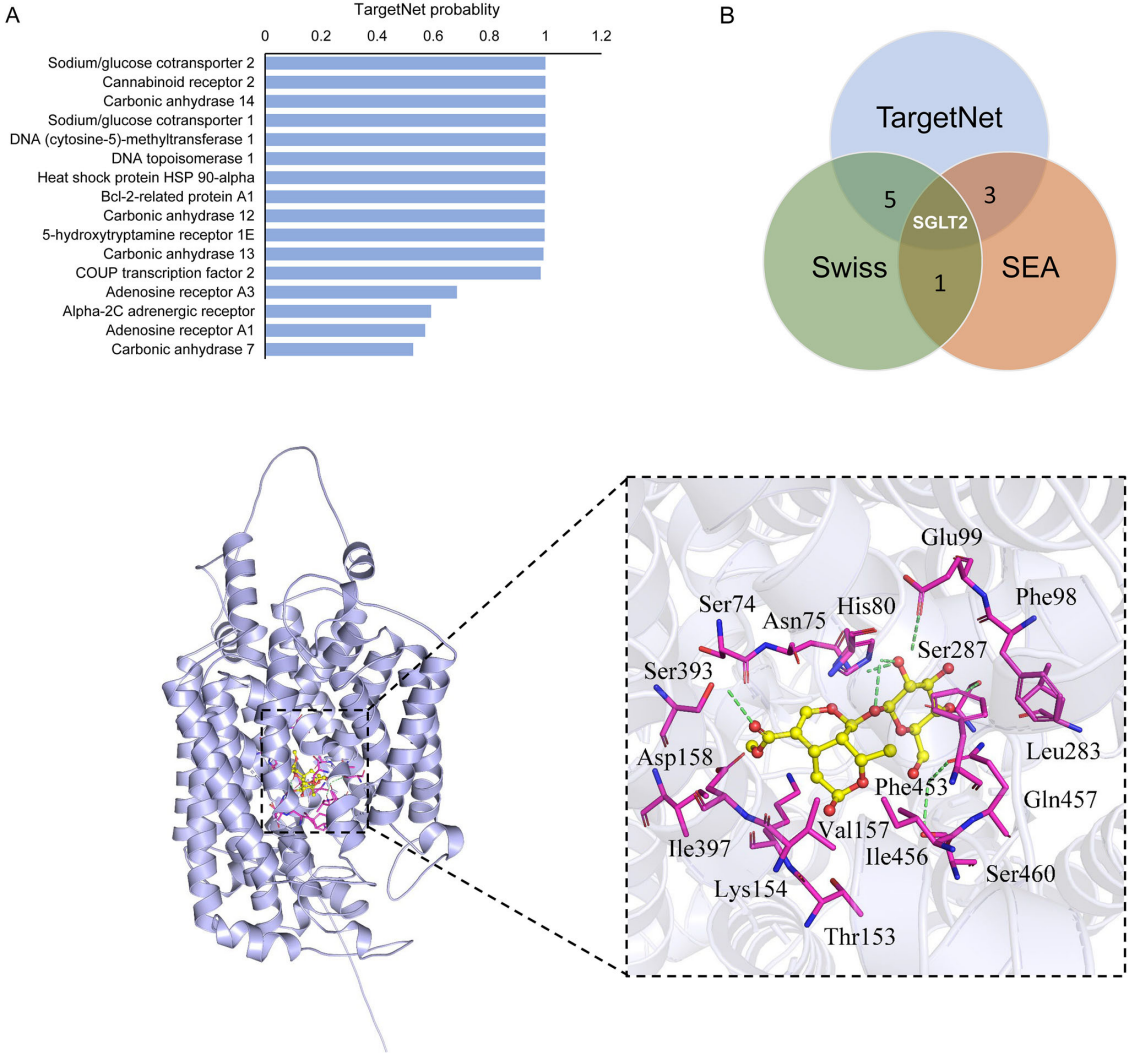
Target prediction and molecular docking of morroniside. (A) The top*-*ranking interacting protein for MORs from the TargetNet server. (B) Venn graph showing the numbers of overlapping targets predicted from TargetNet, SwissTarget and SEA. (C) Molecular docking of MOR in the glucose pocket of SGLT2. (D) Closer view of the binding pocket. Green dashed lines: residues involved in the interactions. Cyan sticks: Rg5; yellow dashed lines: hydrogen bonds; magenta sphere: catalytic zinc ion.

To explore the putative targets of MOR, we performed potential target prediction using TargetNet, SwissTargetPrediction and SEA Prediction servers. In predictions, there were 17 targets in TargetNet (probability >0.3), 29 targets in SwissTarget (probability >0.1), and 21 targets in SEA for MOR actions. Among these, the top-ranking probability for MOR was sodium-glucose cotransporter 2 (SGLT2) in the TargetNet server (Figure 5(A)). We then identified the overlap of targets of MOR among three databases using the Venn graph (Figure 5(B)). As a result, six targets were collected by the TargetNet and SwissTarget databases; three targets were found in both the SwissTarget and SEA databases. Notably, we found that only one putative target, SGLT2, was predicted among all three databases. Therefore, SGLT2 was selected as the potential target of MOR for the following molecular docking analysis.

Using the AutoDock Vina program, we performed a molecular docking analysis of the interaction between MOR and SGLT2. As shown in Figure 4(C), MOR was predicted to bind the SGLT2 hydrophobic pocket with favourable binding scores (–7.9 kcal/mol). Ser460, Gln457, Ser287, Glu99, Asn75, His80 and Ser393 were found to interact with the hydrogen in MOR, while hydrophobic interactions were found within the side chains from Ser74, Asn75, Phe98, Leu283, Phe453, Ile456, Val157, Thr153, Lys154, Ile397 and Asp158 residues of SGLT2 (Figure 5(C)). These binding sites shared several key residues with sugar ligand binding, including Asn75, His80, Phe98, Glu99 and Gln457, which were the critical sites in the glucose pocket. All these predictions suggested that MOR might fit into the glucose pocket of SGLT2.

### SGLT2 inhibitor attenuated the effects of morroniside on OP and osteoblast proliferation

**Figure 6.**
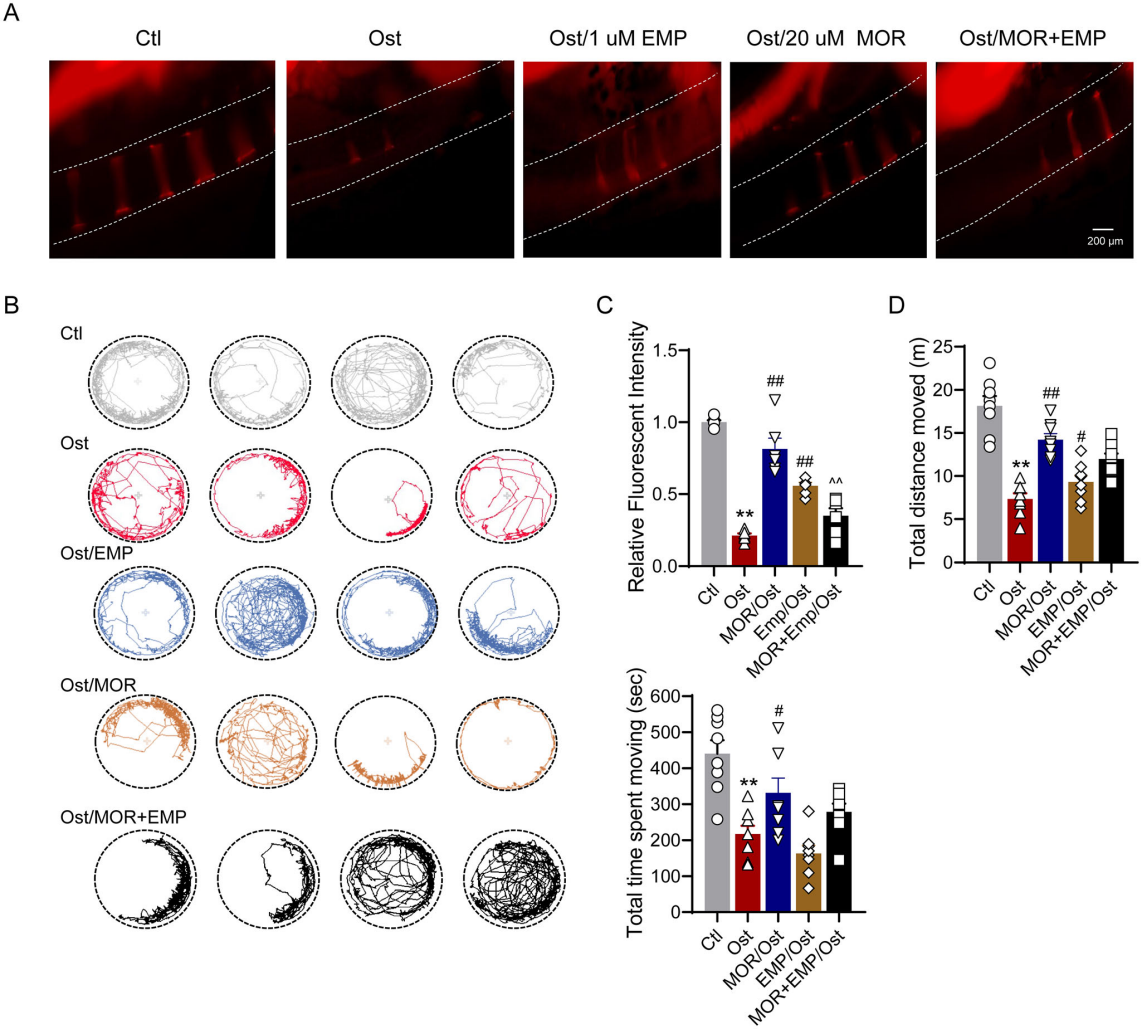
SLC5A2 inhibitor attenuates the effects of morroniside on OP zebrafish. Representative fluorescence images of normal and MOR-treated zebrafish. (A) Representative fluorescence images of the normal (Nor), OP (Ost), EMP, MOR and MOR + EMP treatment groups. (B) The locomotive trajectory of all groups in 20 min. (C) Quantification of the fluorescence intensity of the spinal cord column. Quantification of the total distance moved (D) and total time spent moving (E) in all groups. **p< 0.01 vs. Ctl; ^#^*p*< 0.05, ^##^*p*< 0.01 vs. Ost; ^^^^*p*< 0.01 vs. MOR. *n* = 6.

**Figure 7.**
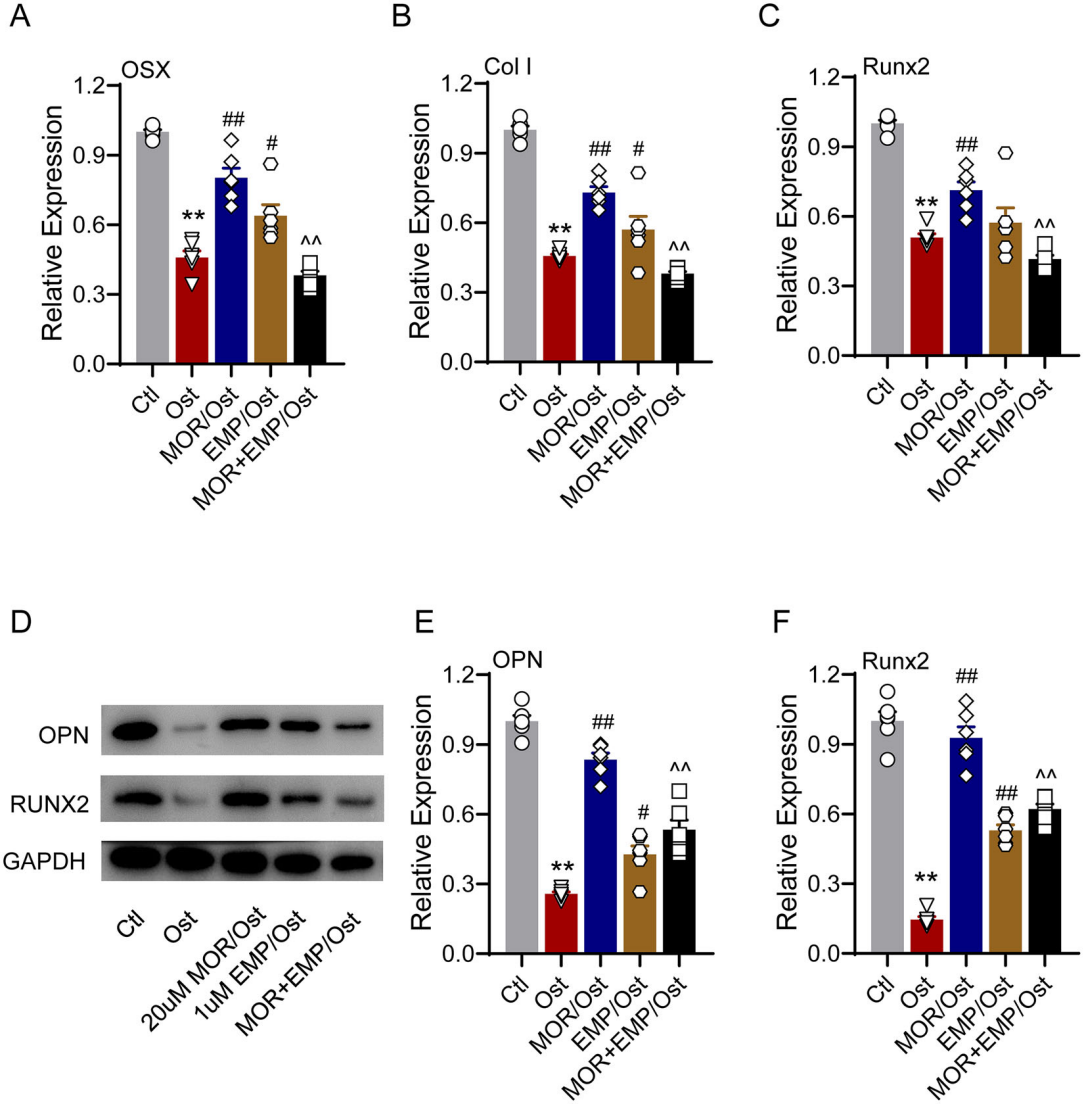
SLC5A2 inhibitor attenuates the effects of morroniside on osteoblastogenesis markers in zebrafish. RT-PCR quantification of osteogenic differentiation marker genes OSX (A), Col I (B) and Runx2 (C) in all groups. (D) Runx2, OPN and GAPDH protein levels were detected by Western blotting in zebrafish. Western blotting quantification of OPN (E) and Runx2 (F). **p< 0.01 vs. Ctl; ^#^*p*< 0.05, ^##^*p*< 0.01 vs. Ost; ^^^^*p*< 0.01 vs. MOR. *n* = 6.

**Figure 8.**
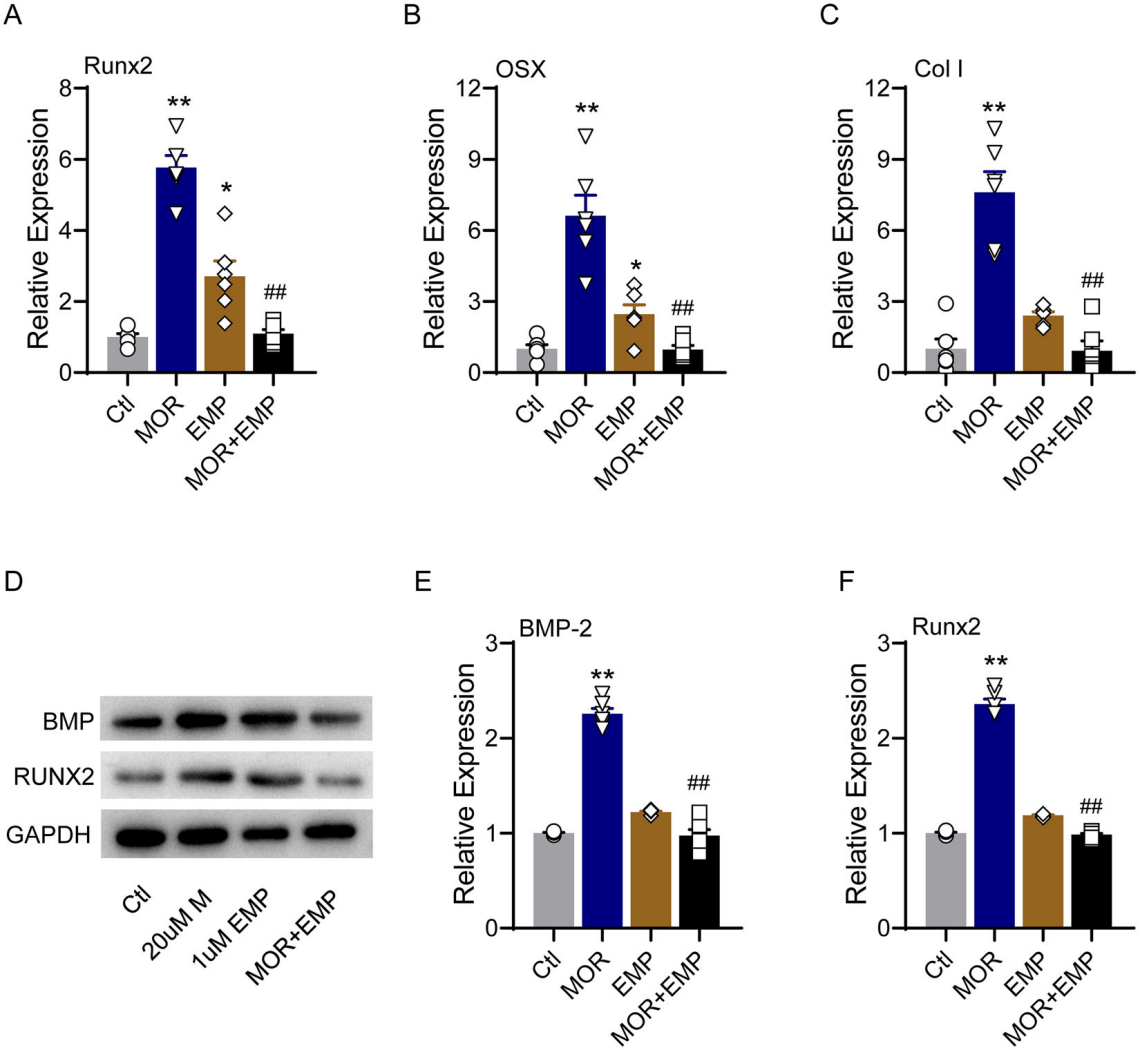
SLC5A2 inhibitor attenuates the effects of morroniside on osteoblastogenesis markers in MC3T3-E1 cells. RT-PCR quantification of osteogenic differentiation marker genes Runx2 (A), OSX (B) and Col I (C) in all groups. (D) Runx2, BMP-2 and GAPDH protein levels were detected by Western blotting in MC3T3-E1 cells. Western blotting quantification of BMP-2 (E) and Runx2 (F). *p< 0.05, **p< 0.01 vs. Ctl; ^##^*p*< 0.01 vs. MOR. *n* = 6.

To confirm whether the pharmacological basis of MOR was associated with SGLT2 binding, we evaluated the effect of the specific SGLT2 inhibitor empagliflozin (EMP), which preferentially binds to glucose pocket sites on the anti-OP performance of MOR. In the glucocorticoid-induced OP zebrafish model, 1 µM EMP was combined with 20 µM MOR interventions for five days. The bone staining and behavioural trajectory results revealed that 20 µM MOR significantly increased osteoporotic zebrafish bone mass (Figure 6(A,C)) and locomotor activity (Figure 6(B)). However, the combination of these two drugs significantly decreased the anti*-*OP effects compared to the MOR groups (Figure 6(C,D)). In addition, combining these two drugs markedly attenuated MOR's promotive effects on osteogenic gene expression in the OP zebrafish model and cells (Figures 7 and 8).

## Discussion

Long-term use of glucocorticoids is the most common cause of secondary OP, resulting in reduced bone mass and altered bone microarchitecture. Unfortunately, current OP interventions are inadequate to treat glucocorticoid*-*induced OP. Therefore, there is an urgent need to investigate alternative therapeutic methods for glucocorticoid*-*induced OP. MOR is one of the major iridoid monoterpenoid glycosides of CF and has been shown to have therapeutic potential for OP (Esen and Long 2014). Using ovariectomized (OVX) mice as a postmenopausal OP model, Lee et al. (2021) found that MOR promoted osteoblast activity and inhibited osteoclast function, suggesting that Mor treatment improved postmenopausal OP. However, the therapeutic effects of MOR on glucocorticoid*-*induced secondary OP and the molecular mechanisms of its action have not been studied. The current study is the first to investigate the anti-OP effects of MOR in glucocorticoid*-*induced OP. Our finding proved that MOR promoted osteoblastic proliferation and effectively ameliorated bone loss in the glucocorticoid*-*induced OP zebrafish model. Furthermore, we found that MOR may exert anti-OP effects by interacting with SGTL2.

Glucose homeostasis plays a crucial role in bone formation and osteogenic differentiation (Esen and Long 2014). Disrupting glucose homeostasis can lead to increased adipogenesis and adipokines, producing severe adverse effects on bone health (Cipriani et al. 2020). The primary mode of glucose utilization in bone tissue is aerobic glycolysis which is stimulated by the parathyroid hormone (PTH) and Wnt pathways to promote bone formation (Wei et al. 2015). The increased aerobic glycolysis in response to Wnt signalling is related to the reduced nuclear levels of both citrate and acetyl-CoA, which is consistent with suppressing adipogenic and chondrogenic transcription factors in osteoblasts (Karner et al. 2016). Hyperglycaemia can stimulate the nonclassical Wnt/protein kinase C pathway and upregulate peroxisome-activated receptor γ (PPARγ), leading to increased adipogenesis and bone loss. It also inhibits the expression of an osteogenic gene, Runx2 (Wei et al. 2015), which is a crucial regulator for osteoblasts maturation and MSCs differentiation (Komori 2019). In addition, the expression of RUNX2 is also regulated by the Wnt/β-catenin signalling pathway in processes such as bone formation and reconstruction (Zhang et al. 2022).

On the other hand, ROS production in anaerobic glycolysis also inhibits osteogenic differentiation and bone formation (Gregorio et al. 1994). Glucose transporters can form a feedback mechanism with Runx2 in osteoblast cell lines, determining the onset of osteoblast differentiation during embryonic development and bone formation and reconstruction throughout the life cycle (Lee et al. 2017). A previous study showed that MOR improved high glucose-induced osteogenic differentiation and alleviated bone loss in a diabetic OP model via the activation of the Glo1pathway (Sun et al. 2020). Our results showed that MOR might bind to a glucose transporter, SGLT2. Given the critical role of glucose homeostasis in osteogenic differentiation, we hypothesized that MOR might improve OP by interacting with glucose transporters. However, these effects of MOR were partially abolished by EMP, a specific SGLT2 inhibitor, suggesting that other mechanisms were involved in this process.

Recent studies have shown that MOR prevents chondrocyte apoptosis and pyroptosis by inhibiting the NF-kappaB signalling pathway (Yu et al. 2021). The nuclear factor-kappa B (NF-κB) signalling pathway in osteoclast precursor cells is mainly responsible for the maturation of osteoclasts and the promotion of their bone resorption (An et al. 2019). Additionally, MOR inhibited autophagy in osteoarthritis through the PI3K/mTOR pathway (Xiao et al. 2020). In addition, MOR showed a neuroprotective effect by inhibiting the release of inflammatory factors and reducing oxidative stress (Zeng et al. 2018; Tang et al. 2020). Moreover, MOR promoted angiogenesis in acute myocardial infarction rats through the VEGFA/VEGF receptor 2 signalling pathway (Liu et al. 2020). Interestingly, our finding reveals that MOR could also interact with SGLT2 to promote osteoblast proliferation and differentiation by regulating glucose homeostasis. However, MOR's pathways to osteoblast proliferation, differentiation and bone formation need further investigation and validation.

We acknowledge that the biological effects of MOR on the transcriptional level are not completely clear at present. Therefore, in a future study, we intend to investigate the transcriptomic changes following MOR treatment using the high-throughput gene expression profiling technique. In addition, zebrafish is a well*-*established animal model for *in vivo* screening drugs that promote bone development (Busse et al. 2020). It shares many similarities but distinct differences in their anatomy, phylogenetic information and physiological properties compared to humans. Another limitation in our study is the lack of confirming the effects of MOR in the bone marrow-derived mesenchymal stem cells (MSCs). Therefore, our study cannot completely rule out the possibility of differences between osteoblastic precursors and osteoblast cells regarding the responses to MOR. Although the MC3T3-E1 cell line is one of the most common osteoblast-like cell lines for studying osteoblast proliferation and differentiation, further studies using MSCs and murine models are required to validate the present results and to develop MOR into a potential therapeutic agent for glucocorticoid induced-OP. Moreover, limited by our cell line and zebrafish model, we did not study MOR’s effect on glucose metabolism in osteoblasts and bone tissue, nor did we measure the changes in intracellular glucose concentration. Therefore, the specific mechanism of the effect of MOR on glucose production in osteoblasts needs to be studied in the near future.

## Conclusions

This study demonstrated that MOR application promoted osteoblast proliferation, differentiation and bone formation. Importantly, MOR may exert these effects by interacting with SGLT2. Therefore, we provided novel insights into the molecular mechanisms of MOR against OP. MOR-SGLT2 may be a potential drug candidate and therapeutic target for preventing and treating glucocorticoid induced-OP.

## Data Availability

The datasets used during the current study are available from the corresponding author on reasonable request.
